# Brain Structural Alterations in Obsessive-Compulsive Disorder Patients with Autogenous and Reactive Obsessions

**DOI:** 10.1371/journal.pone.0075273

**Published:** 2013-09-30

**Authors:** Marta Subirà, Pino Alonso, Cinto Segalàs, Eva Real, Clara López-Solà, Jesús Pujol, Ignacio Martínez-Zalacaín, Ben J. Harrison, José M. Menchón, Narcís Cardoner, Carles Soriano-Mas

**Affiliations:** 1 Psychiatry Department, Bellvitge University Hospital, Bellvitge Biomedical Research Institute (IDIBELL), Barcelona, Spain; 2 Carlos III Health Institute, Centro de Investigación Biomédica en Red de Salud Mental (CIBERSAM), Barcelona, Spain; 3 Department of Clinical Sciences, School of Medicine, University of Barcelona, Barcelona, Spain; 4 Magnetic Resonance Unit, CRC-Hospital del Mar, Barcelona, Spain; 5 Melbourne Neuropsychiatry Centre, Department of Psychiatry, The University of Melbourne, Melbourne, Australia; University of Minnesota, United States of America

## Abstract

Obsessive-compulsive disorder (OCD) is a clinically heterogeneous condition. Although structural brain alterations have been consistently reported in OCD, their interaction with particular clinical subtypes deserves further examination. Among other approaches, a two-group classification in patients with autogenous and reactive obsessions has been proposed. The purpose of the present study was to assess, by means of a voxel-based morphometry analysis, the putative brain structural correlates of this classification scheme in OCD patients. Ninety-five OCD patients and 95 healthy controls were recruited. Patients were divided into autogenous (n = 30) and reactive (n = 65) sub-groups. A structural magnetic resonance image was acquired for each participant and pre-processed with SPM8 software to obtain a volume-modulated gray matter map. Whole-brain and voxel-wise comparisons between the study groups were then performed. In comparison to the autogenous group, reactive patients showed larger gray matter volumes in the right Rolandic operculum. When compared to healthy controls, reactive patients showed larger volumes in the putamen (bilaterally), while autogenous patients showed a smaller left anterior temporal lobe. Also in comparison to healthy controls, the right middle temporal gyrus was smaller in both patient subgroups. Our results suggest that autogenous and reactive obsessions depend on partially dissimilar neural substrates. Our findings provide some neurobiological support for this classification scheme and contribute to unraveling the neurobiological basis of clinical heterogeneity in OCD.

## Introduction

In recent years there has been an increasing interest in studying the clinical heterogeneity of obsessive-compulsive disorder (OCD) [1]–[3]. In particular, the so-called multidimensional model [4], [5] has attempted to summarize OCD in a few temporally stable dimensions that may co-exist within individual patients. Reports comparing patients with different symptom profiles have identified differences in their sociodemographical and clinical features, as well as neurobiological correlates. For instance, in symptom provocation studies, relatively distinctive patterns of brain activity have been associated with the presentation of stimuli representing discrete OCD symptom dimensions, such as aggression/checking and contamination/cleaning symptoms [6]–[8].

Although studies of brain structural alterations in OCD have resulted in a mostly consistent pattern of findings [9]–[11], the assessment of such alterations in relation to specific symptoms or illness subtypes has provided mixed results. Specifically, while in some reports aggressive/checking symptoms were associated with volume alterations in temporolimbic regions, including the amygdala [12], [13], in others this dimension was associated to volume changes in the insula and putamen, among other areas [14]. Similarly, while in some assessments contamination/cleaning and symmetry/ordering symptoms were associated with volume reductions in the dorsal caudate and the sensorimotor cortex, respectively [13], in others such symptoms did not show any significant correlation with brain anatomy [14].

Despite such discrepant findings may be partially explained by the use of different scales to assess symptom dimensions (e.g., the Padua Inventory [15] vs. the Dimensional Yale-Brown Obsessive-Compulsive Scale [16]), it is also true that although the multidimensional approach appropriately accounts for the heterogeneity of OCD symptoms, it may be probably less adequate to describe clear-cut anatomical patterns associated with each clinical subtype, mainly because patients typically score in more than one dimension. In this sense, there have been some attempts to classify OCD symptoms into less overlapping categories, which may well allow us to achieve a less ambiguous identification of the neurobiological underpinnings of clinical heterogeneity in OCD. Nevertheless, the neuroanatomical correlates of such approaches have not been extensively investigated so far.

One such categorical classification was proposed by Lee and Kwon [17], [18], who separated OCD subjects in patients with autogenous and reactive obsessions. While this taxonomy also included a third group of patients experiencing a mixture of obsessions, most patients were classified into one of the two main categories. Significantly, the categorization was not exclusively based on the content of the obsessions, since other clinical features were also taken into account, including the egodystonicity of intrusive thoughts, the induced emotional response or the cognitive appraisals and control strategies evoked by such obsessions. The proposal basically classified subjects with aggressive, sexual or religious thoughts as patients with autogenous obsessions, whereas obsessions concerning contamination, mistakes, accidents or asymmetry and disarray were deemed to be of a reactive nature. Autogenous obsessions were described as being more egodystonic, suddenly appearing without clearly perceived triggers. They were typically observed in patients exhibiting avoidance strategies and high ratings on the “control over thoughts” and “over importance of thoughts” dimensions of the Revised Obsessional Intrusion Inventory [19], [20]. By contrast, reactive obsessions were considered to be less egodystonic and evoked by identifiable stimuli. Such patients normally used confrontational strategies and presented a high level of responsibility in their cognitive appraisals.

This classification was reported as stable in a 3-year follow-up study [21], and has been successfully applied to classify the nature of worries in non-clinical samples [22], [23]. Moreover, studies in clinical samples have substantiated the distinction of both these OCD-patient subtypes through the description of between-group differences in sociodemographical and clinical variables and neurocognitive performance. A higher frequency of males and a higher onset age have been reported in patients with autogenous obsessions [24]. Likewise, autogenous patients presented lower comorbidity with dissociative and OCD spectrum disorders [24] and a better response to pharmacotherapy [21] and cognitive therapy [25], albeit they also showed impaired response inhibition [26], [27]. Neurobiological evidence supporting this classification scheme has also been reported, although further evidence is warranted. Specifically, decreased *N*-acetyl-aspartate (NAA) levels in the limbic medial temporal lobe have been described in autogenous patients, a finding that was partially reversed after a 16-week fluoxetine treatment [28].

The aim of the present study was to assess differences in brain anatomy between patients with primary autogenous and reactive obsessions by means of an exploratory voxel-wise, whole-brain analysis of structural magnetic resonance images (MRI). Additionally, to obtain a reference measurement, both groups were compared with a sample of healthy volunteers of similar age and gender distribution. In addition to substantiating the classification proposed by Lee and Kwon, the identification of anatomical patterns of alteration specifically associated with each category may further expand our knowledge about the neurobiological basis of clinical heterogeneity in OCD.

## Materials and Methods

### Ethics Statement

Written informed consent was obtained from all subjects after a complete description of the study, which was performed in accordance with the Declaration of Helsinki and approved by Bellvitge Hospital’s ethical committee.

### Subjects

A total of 192 subjects were scanned, although two patients were excluded from the final sample as they reported obsessions of both an autogenous and reactive nature. The study sample was thus made up of 95 OCD outpatients (46 females, mean age ± SD = 33.85±9.33 years) and 95 healthy controls (40 females, mean age ± SD = 33.92±10.53). Subjects with OCD were recruited from the OCD Clinic and Research Unit of Bellvitge University Hospital (Barcelona, Spain). Patients were interviewed by two psychiatrists with extensive experience in OCD (P.A and C.S), and the Structured Clinical Interview for DSM-IV Axis I Disorders-Clinician Version (SCID-IV) was used to confirm the diagnosis. All patients met DSM-IV criteria for OCD for at least one year, and had been stably medicated for at least a 3-month period prior to the MRI. Exclusion criteria included: 1) age under 18 or over 65, 2) presence or past history (in the previous six months) of psychoactive substance abuse or dependence, 3) mental retardation, 4) neurological disease comorbidity except tic disorder, 5) present or past history of psychotic disorders, 6) presence or past history of any other severe medical condition, and 7) any contraindication to MRI scanning. Comorbidity with other Axis I disorders was not considered an exclusion criterion provided that OCD was the main diagnosis and the reason for seeking medical assistance.

Healthy controls were recruited from the same sociodemographic environment. Prior to inclusion, each control participant underwent the Structured Clinical Interview for DSM-IV (non-patient version) to exclude presence or past history of any psychiatric disorder. The other exclusion criteria were the same used for OCD patients’ selection.

### Clinical Assessment

Sociodemographic and clinical information was obtained from a semi-structured interview. OCD severity was assessed using the clinician-administered version of the Yale-Brown Obsessive-Compulsive Scale (Y-BOCS) [29] (the Spanish version was used [30]), and depressive symptoms were assessed by means of the 17-item Hamilton Depression Rating Scale (HDRS) [31] (the Spanish version was used [32]). OCD patients were classified into the autogenous and reactive groups based on the primary obsession referred to by the patients following two independent clinical interviews conducted by two psychiatrists with a wide experience in OCD (P.A. and C.S.), who subsequently reached a consensus on each patient’s final classification. Specifically, different clinical features were assessed to obtain a complete depiction of the symptom profile of each participant, such as obsession content, as assessed by the Y-BOCS Symptom Checklist [29], [30], the egodystonicity and perceived rationality of the obsessions, the presence of trigger stimuli, and the cognitive appraisals and avoidance strategies displayed by the patients. To ensure the reliability of the classification process, we developed a classification template to contribute to patient categorization (see Figure S1), and intra- and inter-rater reliabilities were estimated.

The autogenous group was made up of patients with primary aggressive, sexual and moral/religious obsessions that were perceived as highly egodystonic and unrealistic with either no clear triggers or triggers only symbolically related to their obsessions. Such patients typically display avoidance strategies and refrain from confronting their obsessions. By contrast, the reactive group included patients with primary obsessions concerning contamination, mistakes, accidents, asymmetry or disarray. Such obsessions were perceived as more realistic and less egodystonic and were associated to clear trigger stimuli. Patients with reactive obsessions typically display confrontational strategies in response to their obsessions (e.g. washing, checking or counting).

### MRI Acquisition

All images were acquired with a 1.5 T scanner (Signa Excite system, General Electric, Milwaukee, WI, USA) equipped with an eight-channel phased-array head coil. A high resolution T1-weighted anatomical image was obtained for each subject using a 3-dimensional fast spoiled gradient inversion-recovery prepared sequence with 130 contiguous slices in the axial plane (repetition time = 11.8 ms, echo time = 4.2 ms and flip angle = 90°, within a field of view of 30 cm, with a 256×256 pixel matrix and a slice thickness of 1.2 mm). Imaging data were transferred and processed on a Microsoft Windows platform using MATLAB version 7.8 (The Mathworks Inc, Natick, Massachusetts) and Statistical Parametric Mapping software (SPM8; Wellcome Department of Imaging Neuroscience, London, United Kingdom).

### Data Preprocessing

After inspection for the presence of artifacts, images were pre-processed in accordance with a standard protocol involving tissue segmentation, normalization and smoothing. Image segmentation was performed by means of the ‘new segment’ algorithm, as implemented in SPM8. Specifically, after an initial normalization to the Montreal Neurological Institute (MNI) standard space, for each subject we obtained a gray matter image segment, although we discarded final output images from this pre-processing step and reserved the rigidly transformed versions to be used for DARTEL normalization [33]. Thus, with the ‘Create Templates’ function, such images were iteratively matched to a template generated by averaging all individual images to create a series of templates with increasing resolution. Subsequently, native space gray matter images were registered to the highest resolution gray matter template within a high-dimensional diffeomorphic framework. Spatially normalized tissue maps were then modulated by the Jacobian determinants derived from the corresponding flow-fields to restore volumetric information. Finally, images were smoothed with an 8 mm full-width at half-maximum isotropic Gaussian kernel.

### Statistical Analyses

Sociodemographic variables were compared between autogenous and reactive patients and healthy controls by means of one-way ANOVA and X^2^ tests. Furthermore, autogenous and reactive groups were also compared in terms of clinical features using Student’s t and X^2^ tests. Significance threshold was set at p<0.05. The analyses were conducted in SPSS v.20 (SPSS Inc., Chicago, IL).

Voxel-wise regional volumes were compared between groups using SPM8 within the framework of the general linear model. Specifically, we used a one-way ANOVA model to compare autogenous and reactive OCD patients both mutually and with a healthy control group. Age, gender and total gray matter volume were entered as nuisance covariates. In the analyses, statistical significance was established by combining voxel-level and cluster-level significance thresholds. The cluster extent threshold was determined, using the AlphaSim function implemented in the SPM-REST toolbox [34], by means of 5000 Monte Carlo simulations, with a voxel-level significance of p<0.001, a cluster connection radius of 5 mm (SPM default), a gray-matter whole brain mask of 303,754 voxels and the actual smoothing of the data after model estimation. This resulted in a minimum spatial cluster extent (K_E_) of 300 voxels to satisfy a family-wise error (FWE) rate of p<0.05. However, the resulting cluster extent was further adjusted to account for the non-isotropic smoothness of VBM images in accordance with Hayasaka et al. [35].

SPM findings were further characterized in *post-hoc* analyses conducted in SPSS. We extracted the voxel-values from the peak coordinates of the above analyses to assess for differences in all pair-wise comparisons. Such analyses allowed us to characterize regions of gray matter volume difference between autogenous and reactive patients in relation to healthy controls and differences between control subjects and one subgroup of patients in relation to the other group of OCD subjects. Likewise, such voxel-values were correlated, within the OCD groups, against clinical variables such as age at onset, symptom severity or depressive symptoms. Age, gender and total gray matter volume were entered as nuisance covariates and a significance threshold of p<0.05 was used.

## Results

### Sample Characteristics

Sociodemographic and clinical characteristics of patient groups and healthy controls are summarized in Table 1. No differences between patients and healthy controls were observed in age, gender or handedness. Regarding patient distribution into the two OCD groups, 30 patients (31.6%) were classified as having autogenous obsessions and 65 (68.4%) as suffering from reactive obsessions. Two patients, who reported obsessions of both an autogenous and reactive nature, were consequently excluded from any further analysis.

**Table 1:** Sociodemographic and clinical characteristics of the study sample.

	Autogenous (n = 30)	Reactives (n = 65)	Controls (n = 95)	Statistic value^a^ (p value)
***Sociodemographic***				
**Age (y), mean (SD)**	32.23 (9.05)	34.60 (9.43)	33.92 (10.53)	0.583 (0.560)
**Gender (male), n (%)**	20 (66.67)	29 (44.62)	55 (57.9)	4.793 (0.091)
**Handedness (left), n (%)**	3 (11.1)	5 (8.2)	4 (6.6)	0.221 (0.802)
***Clinical***				
**Age at onset** b **(y), mean (SD)**	17.37 (6.67)	22.92 (7.94)	–	−3.327 (0.001)
**OCD family history** c **, n (%)**	5 (16.67)	6 (9.38)	–	1.051 (0.305)
**Stressful life event, n (%)**	10 (33.33)	32 (49.23)	–	2.103 (0.185)
**Tic disorder, n (%)**	5 (16.67)	9 (13.85)	–	0.130 (0.760)
**YBOCS score, mean (SD)**	25.80 (4.24)	27 (6.07)	–	−1.262 (0.210)
**HDRS score, mean (SD)**	12.87 (4.26)	12.69 (4.92)	–	0.163 (0.871)

HDRS, Hamilton Depression Rating Scale; OCD, Obsessive-Compulsive Disorder; SD, Standard Deviation; y, years; YBOCS, Yale-Brown Obsessive Compulsive Scale.

a Statistic value corresponds to ANOVA’s F or t-student test for continuous variables and chi-square test for categorical variables.

b Age at onset was defined as the age when symptoms became a significant source of distress and interfered with the patient’s social functioning.

c A positive familial history of OCD was considered when a first or a second order relative had been formally diagnosed by a psychiatrist.

We estimated the intra- and inter-rater reliability of such a classification, on the one hand, by instructing both the classifying psychiatrists to independently re-assess a random sub-sample of 47 patients, and, on the other hand, by instructing two psychiatrists from outside the OCD unit to classify all the study participants on the basis of the same classification template used for the original classification (see Figure S1). We obtained full agreement in both cases.

Importantly, the two groups of patients only differed in terms of the disorder onset age (with autogenous obsession patients showing an earlier age of onset, see Table 1).

### Imaging Analyses

**Figure 1:**
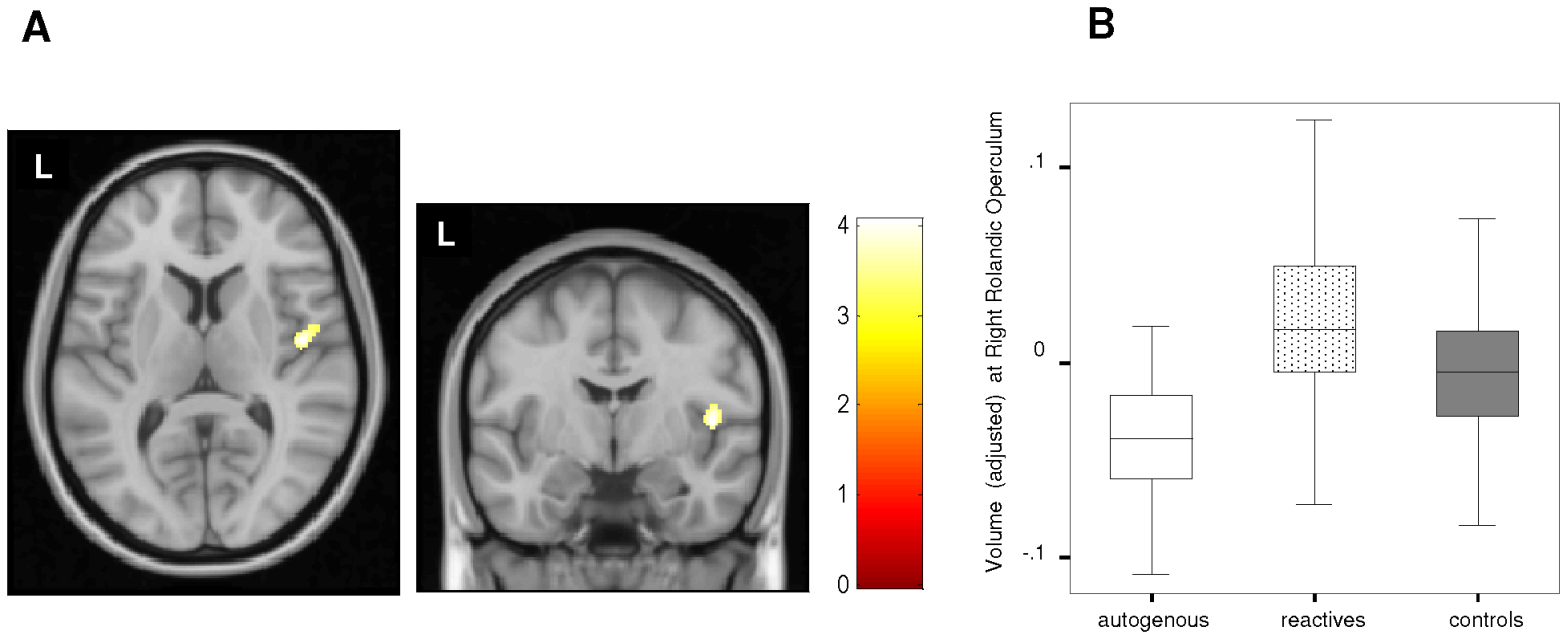
Gray matter volume differences between autogenous and reactive groups. **A. **In comparison to patients with autogenous obsessions, patients with reactive obsessions showed a larger volume in the region of the right Rolandic operculum. Color bar represents t value. L indicates left hemisphere. **B.** Box-plot depicting adjusted GM volume (in imaging units equivalent to volumetric units) corresponding to peak coordinate of the Rolandic operculum in autogenous, reactive and control groups.

In the direct comparison between the two groups of patients, reactive subjects showed a significantly greater gray matter volume in the right Rolandic operculum/posterior insula region (Table 2 and Figure 1, left panel). In a *post-hoc* analysis, we compared the volume of this region between both OCD groups and healthy controls. While autogenous patients showed a lower gray matter volume, reactive patients presented a significantly higher volume (Figure 1, right panel).

**Table 2:** Regions with significant GM volume alterations characterizing the OCD subgroups.

	Peak coordinate (x,y,z)	T	K_E_ (cluster extent)	Anatomical Localization
**REACTIVE>AUTOGENOUS**	45,−7,9	4.06	188(*)	*Right Rolandic Operculum*
**REACTIVE>CONTROLS**	33,−15,0	5.70	1006	*Right Putamen*
	−32,−16,−2	5.38	545	*Left Putamen*
**AUTOGENOUS<CONTROLS**	−56,3,−30	4.03	1436	*Left anterior temporal lobe*

x,y, z coordinates are reported in standard Montreal Neurological Institute (MNI) space.

* This cluster extent reached statistical significance after adjusting for the non-isotropic smoothness of VBM data following Hayasaka et al. (2004) correction.

**Figure 2:**
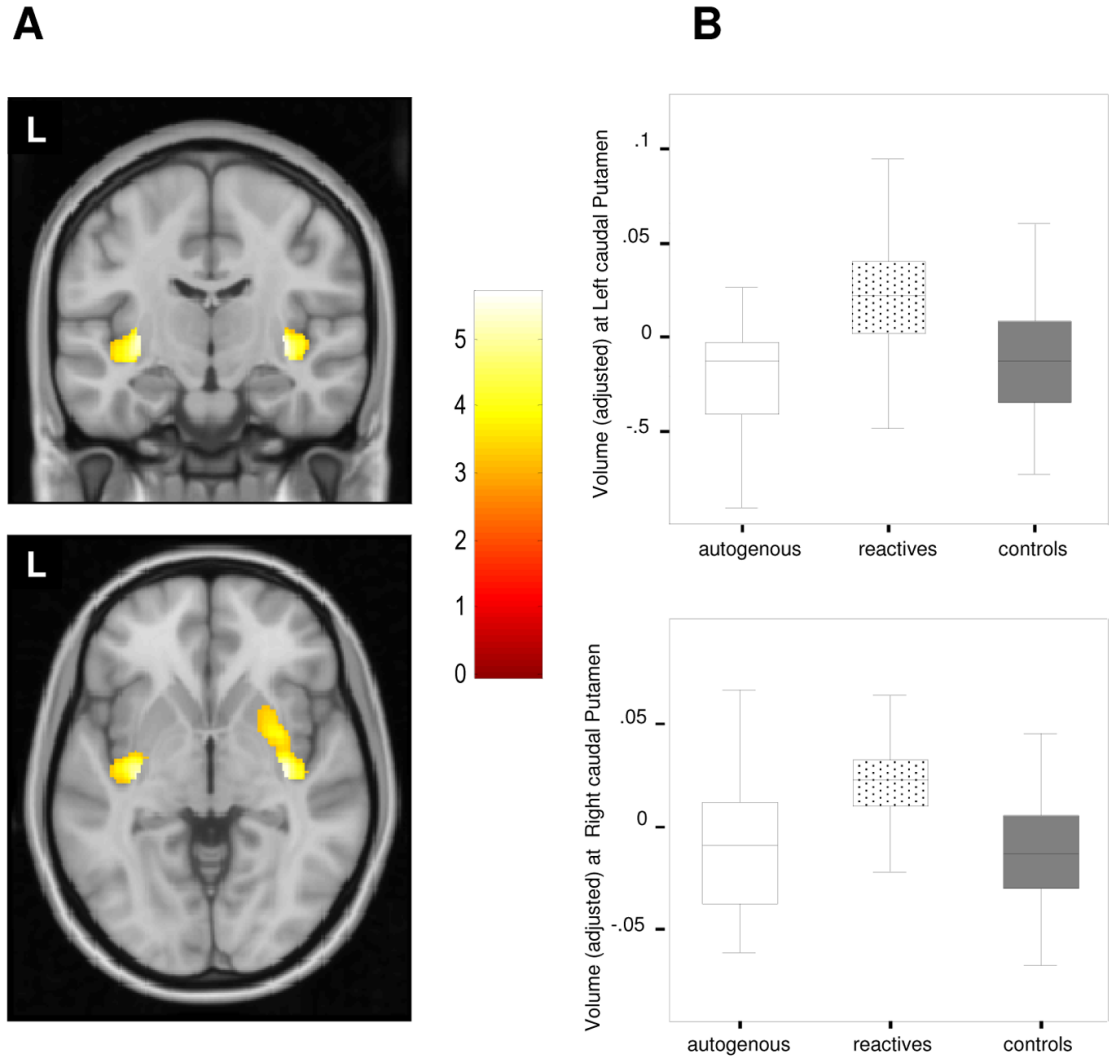
Gray matter volume differences between reactive group and healthy controls. **A. **In comparison to healthy subjects, reactive OCD patients showed a larger caudal putamen (bilaterally). Color bar represents t value. L indicates left hemisphere. **B.** Box-plot depicting adjusted GM volume (in imaging units equivalent to volumetric units) corresponding to peak coordinate in the left caudal putamen (above) and the right caudal putamen (below) in autogenous, reactive and control groups.

We further explored for potential volume differences between the OCD groups by assessing their respective volume changes in relation to healthy controls. Patients with reactive obsessions showed a significantly larger volume of the caudal putamen (bilaterally) (Figure 2, left panel) and a significantly lower gray matter volume in the right middle temporal gyrus (Figure S2, left panel).

**Figure 3:**
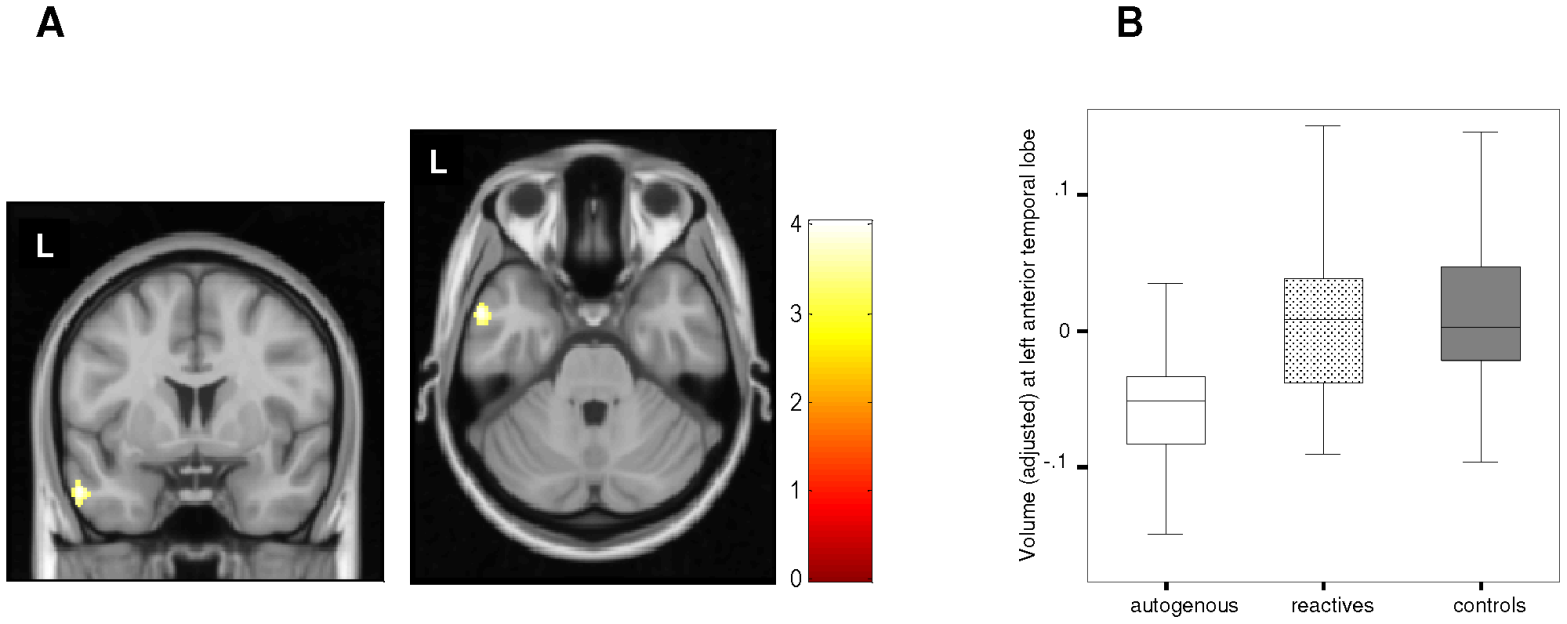
Gray matter volume differences between autogenous group and healthy controls. **A. **In comparison to healthy controls, autogenous OCD patients showed a smaller GM volume in the left anterior temporal lobe. Color bar represents t value. L indicates left hemisphere. **B.** Box-plot depicting adjusted GM volume (in imaging units equivalent to volumetric units) corresponding to peak coordinate in the left anterior temporal lobe in autogenous, reactive and control groups.

By contrast, we observed no significant gray matter volume alterations in patients with autogenous obsessions. However, as such a negative finding was possibly attributable to the lower statistical power of the comparison (in relation to the comparison of a larger group of reactive OCD subjects against controls), the analysis was reassessed at a more lenient voxel-level significance threshold (p<0.01). We did not, however, observe any region of larger gray matter volume in autogenous patients despite the more lenient threshold level (either in the caudal putamen or any other brain region). Conversely, at this particular significance threshold we did observe the same smaller gray matter volume in the right middle temporal gyrus described for reactive patients. Moreover, we also observed a lower gray matter volume in the left anterior temporal lobe, which was not observed in the case of patients with reactive obsessions (Figure 3, left panel).

In post-hoc analyses, we further characterized the above findings for all pair-wise comparisons. As for the caudal putamen, it was bilaterally smaller in patients with autogenous obsessions in comparison to reactive patients, but not in relation to healthy controls (Figure 2, right panel). The right middle temporal gyrus was not significantly different between the two patient groups (Figure S2, right panel). Finally, the left anterior temporal lobe cluster was smaller in autogenous patients in comparison to reactive patients, which did not differ from healthy controls (Figure 3, right panel).

All the above analyses were repeated after adding age at onset, HDRS and YBOCS scores as confounding covariates, with no significant changes in the results.

Finally, correlation analyses were performed between neuroimaging data and the clinical variables of interest (see Table 1) and no significant associations were found.

## Discussion

Our study assessed the regional gray matter volume alterations of OCD patients with autogenous and reactive obsessions. In comparison to autogenous patients, reactive patients showed a larger gray matter volume in the right Rolandic operculum. Also, when compared to healthy controls, patients with reactive obsessions showed a larger caudal putamen. Patients with autogenous obsessions showed a smaller left anterior temporal lobe, albeit at a lower significance threshold. Finally, we also observed a smaller gray matter volume in the right middle temporal gyrus, although it was observed in both OCD groups.

The proportion of patients with autogenous and reactive obsessions in our sample reflects a naturalistic distribution, similar to what has previously been reported [21], [25]. Although in such studies a third group of patients with a mixed content of obsessions was described, in our sample only two patients were classified in the mixed subtype. In this sense, it is important to highlight that our classification was based on the primary obsession referred to by the patients, as in previous studies [18], [25], [26]. By contrast, other authors have preferred to base the classification of OCD patients on the overall obsessional content [28]. Such a methodological difference may partially account for the between-study discrepancies in the proportion of mixed-subtype patients. Nevertheless, previous studies have also shown high agreement between both classification approaches [26]. Regarding the clinical characteristics of our OCD groups, patients with autogenous obsessions reported an earlier age at onset. Such a result is at odds with other reports showing an earlier onset in reactive patients [24] and, indirectly, with reports of longer illness duration in reactive patients [25]. Nonetheless, the heterogeneous results obtained in the few previous reports comparing clinical variables between autogenous and reactive patients, such as Lee et al. [18], who did not observe any differences in illness duration, prevent us from elucidating the possible causes of such discrepancies. Speculatively, however, they may partially relate to between-study differences in the definition of disorder onset, in combination with the difficulty of establishing a precise age of onset by means of retrospective assessment.

### Gray Matter Volume Differences between Groups

In comparison to autogenous patients, reactive patients showed a significantly greater gray matter volume in the right Rolandic operculum, extending to the adjacent posterior insular cortex. Specifically, the region of between-group differences comprises the areas OP3 and OP4 of the secondary somatosensory cortex, approximately corresponding to Broadman’s area 43 [36]. Interestingly, this region has been related to impulsivity features. In a recent study of healthy adolescents, Moreno-López et al. [37] described a negative correlation between OP4 volume and positive urgency (a subtype of impulsive behavior), and structural alterations in the region have been described in children suffering from attention deficit-hyperactivity disorder [38]. Although compulsivity and impulsivity have been typically regarded as opposite concepts, recent approaches would suggest the existence of a considerable degree of mutual overlapping [39], [40], and, indeed, the existence of an impulsive OCD subtype has been proposed [41]. It may, therefore, be suggested that a smaller volume in the Rolandic operculum accounts for the impulsive nature of some autogenous patient obsessions (i.e., impulsive phobias). Likewise, autogenous patients have been shown to display an impaired inhibitory control, with difficulties in filtering irrelevant distracters [26].

In parallel, the Rolandic operculum has been associated with the neural mechanisms of tic generation and, more specifically, with the premonitory urges that generate tic behaviors [42]. Although our sample included very few subjects with comorbid tic disorder (see Table 1), subjective experiences similar to those preceding tic behaviors [43], [44] have been described in almost 60% of OCD patients [45]. Such subjective experiences, or sensory phenomena, are characterized by uncomfortable and distressing physical sensations -tactile, muscular or interoceptive- accompanied by urges. Interestingly, the presence of sensory phenomena has particularly been related to the presence of symmetry/ordering symptoms [45], classified here as reactive obsessions. Moreover, in a recent study comparing OCD patients with and without sensory phenomena, the intrusive thoughts classified here as autogenous were less frequently observed in patients with sensory phenomena [46]. The extension of this cluster to the posterior insular cortex, which is involved in the integration of perceptual experiencies, especially those related to somesthesia and skeletomotor perception [47], would also seem to support such a relationship between reactive obsessions and the presence of sensory phenomena. Unfortunately, however, we did not specifically assess the presence of sensory phenomena in the present sample. Nevertheless, such data do provide a framework in which we can interpret the increased right Rolandic operculum volumes observed in patients with reactive obsessions.

### Gray Matter Volume Differences between OCD Subgroups and Healthy Controls

In comparison to healthy controls, we observed a significant larger putamen in reactive patients. Significantly, this larger volume was not observed in patients with autogenous obsessions even at a lower significance threshold and thus the possibility that such a negative finding was due to the lower statistical power of the comparison between autogenous patients and healthy controls was ruled out. Functional and structural alterations in different striatal territories have been described in OCD [10], [48]–[51], and the dorsal putamen has been consistently related to habit formation and the development of stereotyped motor sequences and compulsive behaviors [52]–[54]. Bearing such notions in mind, it is important to note that some classifications have described the existence of a group of “pure obsession” patients [55], who do not show overt compulsive behaviors, but rather a high rate of mental compulsions [56]. Interestingly, such patients typically present obsessions involving forbidden and unaccepted thoughts, classified here as autogenous. Our findings may thus indicate that volume increases in the putamen characterize the group of patients with reactive obsessions, who display a higher incidence of overt compulsive behavior.

Patients with reactive obsessions also exhibited less gray matter in the right middle temporal region, although such a finding was equally observed in patients with autogenous obsessions at a lower significance threshold. Conversely, a smaller volume of the left anterior temporal lobe was specifically observed in patients with autogenous obsessions. While previous studies reported the development of obsessive-compulsive symptoms after temporal pole lesions [57], Van den Heuvel et al. [13] showed a specific association of anterior temporal lobe volumes and harm/checking symptoms that partially overlap with the symptoms classified here as autogenous. Moreover, patients with autogenous obsessions typically display an exacerbated distress when dealing with questions of a moral nature. Anterior temporal lobe alterations may account for such dysfunctional cognitions, as this region has been related to complex cognitive processes such as moral cognition [58], and the interaction between anterior temporal lobe and fronto-mesolimbic activity has been hypothesized to underpin the experience of moral sentiments [59].

According to our data, general OCD populations should normally include a larger proportion of patients with reactive obsessions. It is, therefore, not surprising that putamen alterations have been previously reported in general OCD samples [9], [60], [61]. At the same time, however, it may seem surprising that volume increases in the Rolandic operculum have not previously been documented. Nevertheless, it should be pointed out that, in our post-hoc analysis, autogenous patients presented a significantly smaller volume of this particular brain region, which may well partially compensate the larger volumes of reactive patients when assessing general OCD populations. Likewise, it is noteworthy that we have not described alterations involving other brain regions such as the dorsal-medial prefrontal and the medial and lateral orbitofrontal cortices, which have typically presented alterations in studies assessing general OCD samples [9], [10]. Smaller volumes in these regions may not, therefore, depend on the classification scheme used here and are most probably related to other symptoms. By way of example, the presence of comorbid depression would seem to be particularly important in relation to orbitofrontal alterations [62] and our study groups did not differ in terms of this variable.

Certain limitations apply to the current findings. Firstly, as recruitment was conducted in a specialized OCD clinical unit, the mean severity of our sample was somewhat higher in comparison to other reports. Secondly, patients were undergoing pharmacological treatment during the study period. Nevertheless, no significant effects of antidepressant treatment on brain morphology were detected in a voxel-wise meta-analysis of structural studies in OCD [9]. Finally, given the lack of an objective measurement to identify autogenous and reactive patients, the possibility of a classification bias cannot be ruled out. Be that as it may, the classification was carried out by two expert psychiatrists who reached a consensus as to the nature of each patient’s symptoms.

In summary, the existence of specific structural alterations in patients with autogenous and reactive obsessions provides some neurobiological support for this classification scheme, as proposed by Lee and Kwon [18]. These findings add to emerging evidence from neuroimaging studies that the clinical heterogeneity in OCD can be differentiated in terms of discrete brain systems. Future studies should expand our results by relating the anatomical abnormalities of patients with autogenous and reactive obsessions with specific clinical features, such as impulsivity, sensory phenomena, overt compulsions, or exacerbated moral distress.

## Supporting Information

Figure S1 **Classification template used for OCD patients’ characterization.** This template was intended to assist the psychiatrists in the classification of OCD patients according to their primary obsessions.
(TIF)

Figure S2 **Gray matter volume differences between OCD subgroups and healthy controls. A.** In comparison to healthy controls, reactive OCD patients showed a smaller GM volume in the right middle temporal gyrus. Color bar represents t value. L indicates left hemisphere. **B.** Box-plot depicting adjusted GM volume (in imaging units equivalent to volumetric units) corresponding to peak coordinate in the right middle temporal gyrus in autogenous, reactive and control groups.
(TIF)
