# Supplementary figures and images for: Brain Structural Alterations in Obsessive-Compulsive Disorder Patients with Autogenous and Reactive Obsessions
Source: PLoS One. 2013 Sep 30;8(9):e75273. doi: 10.1371/journal.pone.0075273 (PMC3787080; doi:10.1371/journal.pone.0075273)

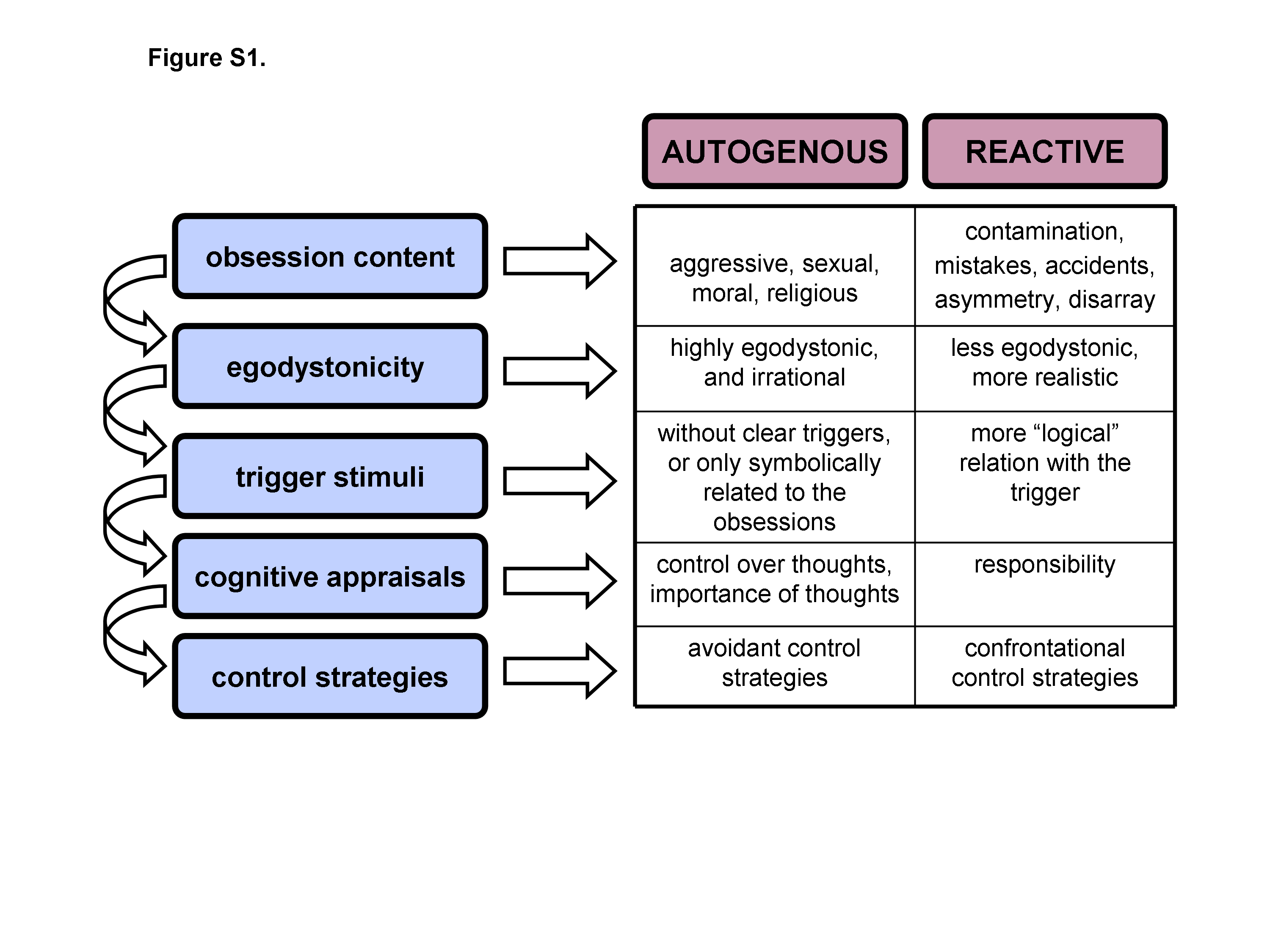

Supplement: Figure S1 — Classification template used for OCD patients’ characterization. This template was intended to assist the psychiatrists in the classification of OCD patients according to their primary obsessions. (TIF) [file pone.0075273.s001.tif]

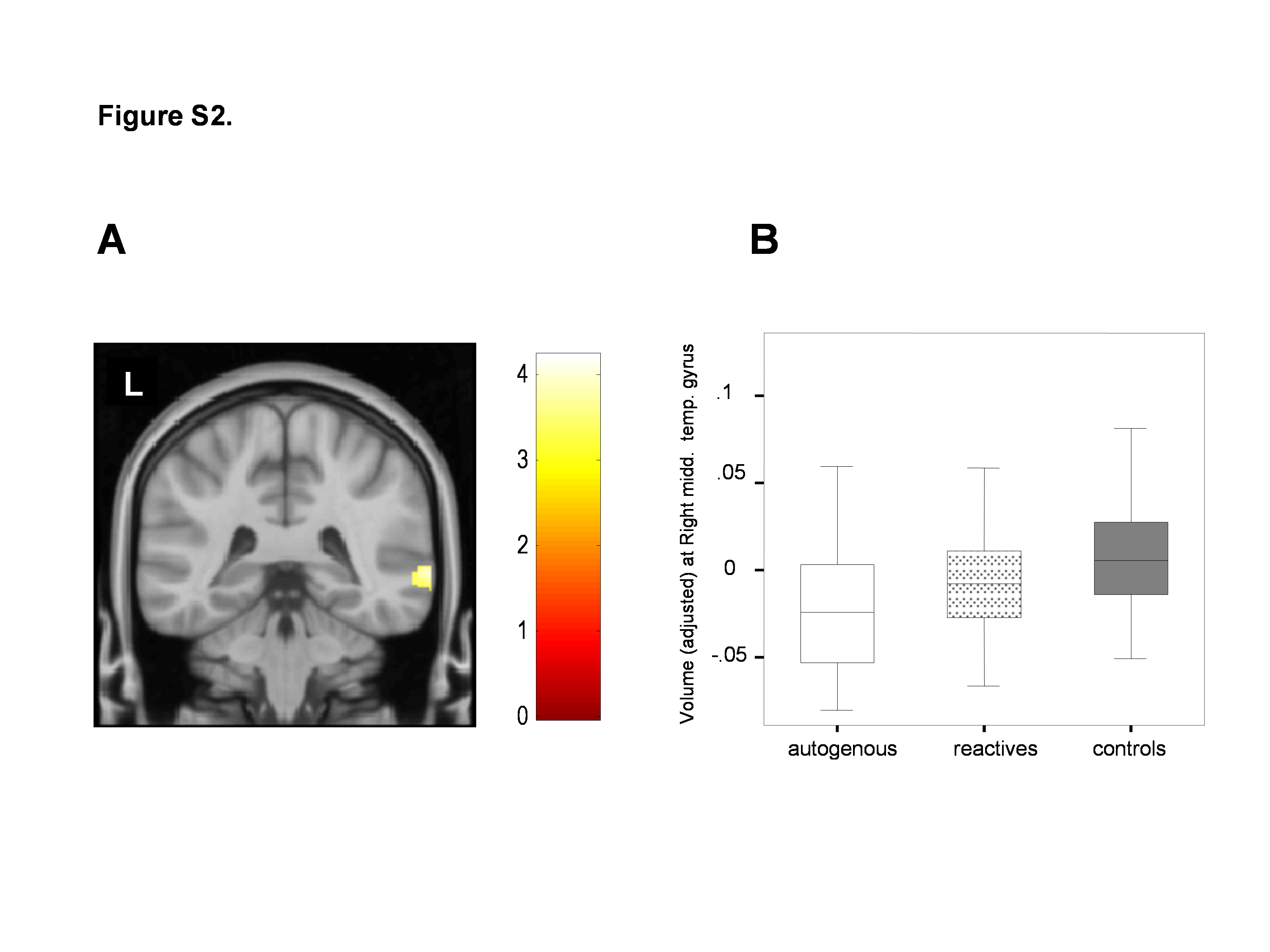

Supplement: Figure S2 — Gray matter volume differences between OCD subgroups and healthy controls. A. In comparison to healthy controls, reactive OCD patients showed a smaller GM volume in the right middle temporal gyrus. Color bar represents t value. L indicates left hemisphere. B. Box-plot depicting adjusted GM volume (in imaging units equivalent to volumetric units) corresponding to peak coordinate in the right middle temporal gyrus in autogenous, reactive and control groups. (TIF) [file pone.0075273.s002.tif]
